# The Capsicum terpenoid biosynthetic module is affected by spider-mite herbivory

**DOI:** 10.1007/s11103-023-01390-0

**Published:** 2023-11-23

**Authors:** Yuanyuan Zhang, Arman B. Kashkooli, Suze Blom, Tao Zhao, Harro J. Bouwmeester, Iris F. Kappers

**Affiliations:** 1 Laboratory of Plant Physiology, Wageningen University, Wageningen, The Netherlands; 2 Present Address: College of Forestry and Landscape Architectures, South China Agricultural University, Guangzhou, China; 3 Present Address: Tarbiat Modares University, Tehran, Iran; 4 Present Address: Laboratory of Molecular Biology, Wageningen University, Wageningen, The Netherlands; 5 Bioscience, Wageningen University & Research, Wageningen, The Netherlands; 6 Biosystematics, Wageningen University, Wageningen, The Netherlands; 7 Present Address: Northwest Agriculture and Forestry University, Xi’an, China; 8 Swammerdam Institute for Life Sciences, University of Amsterdam, Amsterdam, The Netherlands

**Keywords:** *Capsicum annuum*, Terpene synthases, Terpenoid biosynthetic module, Terpenoid metabolites, Two-spotted spider mite herbivory

## Abstract

In response to herbivory, *Capsicum annuum* leaves adapt their specialized metabolome that may protect the plant against herbivore feeding either directly or indirectly through volatile metabolites acting as cues for natural enemies of the herbivore. The volatile blend of spider-mite infested leaves differs from non-challenged leaves predominantly by a higher contribution of mono- and sesquiterpenes. In addition to these terpenoids released into the headspace, the terpenoid composition of the leaves alters upon herbivory. All this suggests an important role for terpenoids and their biosynthetic machinery in the defence against herbivory. Here, we show that the *C. annuum* genome contains a terpene synthase (*TPS*) gene family of 103 putative members of which structural analysis revealed that 27 encode functional enzymes. Transcriptome analysis showed that several *TPS* loci were differentially expressed upon herbivory in leaves of two *C. annuum* genotypes, that differ in susceptibility towards spider mites. The relative expression of upstream biosynthetic genes from the mevalonate and the methylerythritol phosphate pathway also altered upon herbivory, revealing a shift in the metabolic flux through the terpene biosynthetic module. The expression of multiple genes potentially acting downstream of the *TPSs*, including cytochrome P450 monooxygenases, UDP-glucosyl transferases, and transcription factors strongly correlated with the herbivory-induced *TPS* genes. A selection of herbivory-induced TPS genes was functionally characterized through heterologous expression and the products that these enzymes catalysed matched with the volatile and non-volatile terpenoids induced in response to herbivory.

**Supplementary Information:**

The online version contains supplementary material available at 10.1007/s11103-023-01390-0.

## Key message

Annotation of the *Capsicum annuum* terpene synthase gene family links terpene metabolism to the formation of volatile and non-volatile terpenoids important for direct and indirect defence.

**Supplementary Information:**

The online version contains supplementary material available at 10.1007/s11103-023-01390-0.

## Introduction

Terpenoids represent a large class of chemical compounds with an extensive diversity in chemical structures and a wide range of functions. They are produced by plants, fungi, bacteria, and insects. In plants, terpenoids harbour various phytohormones involved in growth regulation, including gibberellins, abscisic acid, strigolactones and cytokinins as well as metabolites that are involved in important physiological processes such as membrane stabilization (sterols), photosynthesis (carotenoids, the phytol side chain of chlorophyll and plastoquinone) and respiration (ubiquinone) (Tholl and Lee 2011). However, a substantially larger number of terpenoids contribute to broader physiological and ecological functions, including plant defence and plant communication (Singh and Sharma 2015).

Terpenoids are formed from C5 isoprene precursors, isopentenyl diphosphate (IPP) and its isomer dimethylallyl diphosphate (DMAPP) that are produced from the mevalonate pathway (MVA) in the cytosol/peroxisomes or from the methylerythritol phosphate pathway (MEP) in the plastids (Vranová et al. 2013). The condensation of DMAPPs with varying numbers of IPP units is catalysed by prenyl diphosphate synthases to synthesize geranyl diphosphate (GPP) (*trans*-configuration), or neryl diphosphate (NPP) (*cis*-configuration), depending on the prenyl diphosphate synthase, *trans*- or *cis*-farnesyl diphosphate (FPP) and geranylgeranyl diphosphate (GGPP) (Takahashi and Koyama 2006; Wang and Ohnuma 2000). These prenyl diphosphates are substrate for plant terpene synthases (TPSs)—monoterpene synthases, sesquiterpene synthases and diterpene synthases—generating monoterpenes (C10), sesquiterpenes (C15) and diterpenes (C20), respectively (Singh and Sharma 2015).

Part of the structural diversity in the terpenoids is obtained through variation in cyclization and rearrangement reactions of the prenyl diphosphate substrates. After formation of the carbocation usually a multitude of carbocation attacks and/or quenching through water, double bond rearrangements and hydride shifts occur, which is why a single TPS enzyme can sometimes generate multiple products from one substrate (Degenhardt et al. 2009). The structural diversity in these TPS products is often further increased through the activity of cytochrome P450s (Bathe and Tissier 2019), reductases, dehydrogenases methyl transferases (Piechulla et al. 2020; Lemfack et al. 2021) and glycosyl transferases (Boutanaev et al. 2015; Kurze et al. 2022) to yield an enormous variety of different terpenoid products. The myriad of terpenoid structures mirrors the complexity of properties and functions.

TPSs of flowering plants consist of 550–850 amino acids, with a molecular mass of 50–100 kDa and are encoded by gene members of the structurally related *TPS* gene family that probably has a common phylogenetic origin (Aubourg et al. 2002). The number of *TPS* genes within plant species ranges from a single functional terpene synthase gene in the moss *Physcomitrella patens* (Hayashi et al. 2007) to about 20 to 150 in flowering plant species (Chen et al. 2011). Structurally the *TPS* gene family can be divided into seven subfamilies, *TPS-a*, *TPS-b*, *TPS-c*, *TPS-d*, *TPS-e/f*, *TPS-g* and *TPS-h*. The *TPS-a*, *b* and *g* subfamilies are angiosperm specific while the *TPS-d* subfamily is gymnosperm specific and *TPS-h* is specific to the lycopod *Selaginalla moellendorffi* (Li et al. 2012; Chen et al. 2011). *TPS-c* and *e/f* members are present in both angiosperms and gymnosperms; *TPS-c* members encode copalyl diphosphate synthases and *TPS-e/f* members encode kaurene synthases. Most of the *TPS-a* genes encode sesquiterpene synthases while *TPS-b* genes mostly encode monoterpene synthases (Falara et al. 2011). The *TPS-g* subfamily is closely related to *TPS-b* and its members may function in producing acyclic mono-, sesqui-, and diterpene products (Chen et al. 2011).

Terpenoids often play a role in plant–insect interactions, either as attractants of pollinators or as defensive compounds against various herbivores (Kappers et al. 2008). For instance, conifers release volatile mono- and sesquiterpenoids that insects use to distinguish suitable hosts from non-hosts (Keeling and Bohlmann 2006). The colonization by arbuscular mycorrhiza altered terpenoid composition in plants which reinforced both direct and indirect defence against herbivorous insects (Sharma et al. 2017). The non-volatile sesquiterpene lactone argophyllone B reduces the larval mass of the sunflower moth *Homeosoma electellum* in *Helianthus* spp. (Prasifka et al. 2015). In *Nicotiana attenuata*, 17-hydroxygeranyllinalool diterpene glycosides were demonstrated to be effective defensive metabolites to herbivores (Heiling et al. 2010). Besides a direct effect on herbivores, terpenoids play an important role in attracting natural enemies of herbivores. Overexpression of a strawberry nerolidol synthase in Arabidopsis resulted in emission of two new terpenoids (3S)-(*E*)-nerolidol and its derivative (*E*)-4,8-dimethyl-1,3,7-nonatriene (DMNT), which resulted in enhanced attraction of *Phytoseiulus persimilis* predatory mites (Kappers et al. 2005). Transgenic Arabidopsis expressing maize *TPS10* produced more sesquiterpenes and attracted more female *Cotesia mariniventris* parasitoids (Schnee et al. 2006).

The Capsicum genus belongs to the Solanaceae nightshade family and consists of approximately 35 species (Carrizog 2013). Capsicum is native to the Americas and currently cultivated worldwide for their sweet or spicy fruits, which are used as vegetable or spice. Various generalist arthropod species can infest Capsicum resulting in serious yield losses, including western flower thrips (*Franklinella occidentalis*) (Macel et al. 2019), aphids (*Myzus persicae* (Vaello et al. 2018), *Macrosiphum euphorbiae* (Sun et al. 2020) and two-spotted spider mites (*Tetranychus urticae*) (Zhang et al. 2020). The biotic stress-related phytohormone jasmonic acid (JA) is the master regulator of plant defence responses against herbivores (Verma et al. 2016) by mediating the expression of defence-related genes (Rehrig et al. 2014). This includes the biosynthesis of secondary metabolites (Ferrieri et al. 2015; Machado et al. 2017), especially terpenoids, in multiple plant species (Zhang et al. 2009; Hong et al. 2012; Ninkuu et al. 2021). Previously, we showed that JA plays a major role in the early signalling events of spider-mite induced defences in Capsicum and that exogenous application of JA results in notably higher emission of various terpenoids (Zhang et al. 2020). The availability of the genome sequence of a Capsicum inbred line (*C. annuum* var *Zunla)* allows us to predict the Capsicum *TPS* (*CaTPS*) gene family, which will help to understand the mechanism of terpenoid formation and metabolism under herbivory in Capsicum.

Here, we report the genome-wide identification of the *TPS* gene family and the annotation of 27 putative functional *TPS* genes in the *C. annuum* genome. To study how herbivory in general, and specifically spider-mite infestation, affects terpenoid production and emission in Capsicum, we treated plants with JA or spider mites and studied the changes in *CaTPS* gene expression. Several *CaTPS* genes of which the expression was induced by spider mites and/or by JA were subsequently isolated and functionally characterized. Analysis of transcriptional and metabolic changes upon herbivory revealed co-expression patterns between *TPS* genes and upstream biosynthetic genes from the MVA and MEP pathway as well as with genes downstream of the *TPSs* that may be involved in the creation of the structural diversity in herbivory-related Capsicum terpenoids.

## Materials and methods

### Identification of the terpene biosynthetic modules

To identify putative Capsicum *TPS* genes, a BLAST search against the *C. annuum* (Zunla-1) genome database (Qin et al. 2014) was performed using the protein sequence of known terpene synthases from *A. thaliana*, *S. lycopersicum*, *Medicago truncatula*, *Vitis vinifera* and *Picea glauca* (blast hits with e-value lower than e−5). In addition, using the approach described by Hofberger et al. (2015) which combines multiple lines of evidence provided by gene synteny, sequence homology and Hidden Markov Modelling, the *C. annuum* Zunla genome was screened for proteins associated with terpene biosynthesis, thereby covering the complete terpene biosynthetic module. The functional annotation we use in the present study is based on homology with annotated Arabidopsis and tomato genes.

### Phylogenetic tree reconstruction and gene model analysis

Deduced Ca*TPS* genes were aligned using ClustalX2.1 with standard parameters (www.clustal.org/clustal2) and a phylogenetic tree was built with Maximum Likelihood method in MEGA11 (Tamura et al. 2021). The intron–exon structure of Ca*TPS* genes was determined by comparing the coding sequences and genomic sequences using the Gene Structure Display Server (GSDS2.0) (Hu et al. 2015). Putative plastid transit peptides were predicted using the TargetP1.1 server (Emanuelsson et al. 2000). Motifs were visualized using Jalview (https://www.jalview.org/).

### Plants and arthropods

Capsicum plants [*C. annuum,* varieties Vania (Inbred line, Genotype 8) and Ta Pien Chiao (Land race, Genotype 29)] were grown from seed for 5 weeks in a greenhouse with a photoperiod of 16 h (23/18 °C, 50–60% relative humidity). Plants were still in the vegetative stage when used for experiments. *Nicotiana benthamiana* plants were grown from seed in the same greenhouse. Four-week-old, non-flowering plants were used for experiments and plants were not watered for 2 days prior to infiltrations. Two-spotted spider mites *Tetranychus urticae* were originally obtained from Koppert BV (Berkel en Rodenrijs, The Netherlands) and maintained on *Phaseolus vulgaris* bean plants for multiple generations.

### JA treatment and spider-mite infestation

Five-week-old, non-flowering plants were infested with approximately 300 spider mites per plant by placing similar pieces of heavily-infested bean leaves on top of the Capsicum leaves. For jasmonic acid treatment, plants were sprayed with 100 µM methyl-JA (0.01% Tween-20). Mock-treated plants were sprayed with 0.01% Tween-20. For metabolite and transcript analysis, the 4th and 5th leaf were harvested after different periods of infestation/treatment, immediately flash-frozen and powdered in liquid nitrogen. For each treatment, five biological replicates were generated to allow for transcript and metabolite analysis from the same material.

### Transcript data analysis

Assembly of full length transcripts of genes in the terpene biosynthetic module was performed using the Trinity method (Grabherr et al. 2011) on previously constructed transcriptome libraries from Capsicum plants that were infested with spider mites for 3 days, treated with JA for 6 h and 24 h, or were left untreated using the Illumina HiSeq™ 2500 platform (Zhang et al. 2020). Expression levels as RPKM values were calculated for each sample and the relative contributions of genes in the terpene biosynthetic module were calculated for each experimental condition to estimate shifts in flux through the MEP and MVA pathway. Pearson correlation coefficients were calculated between 17 expressed *TPS*s and transcripts assembled from the RNA sequencing. Correlation coefficients above a threshold of 0.90 (either positive or negative) were used to create a co-expression network visualized using the yGraph Organic layout in Cytoscape v.3.9.1 (https://cytoscape.org/).

### Gene expression

Total RNA was isolated from the same samples as used for endogenous metabolite analysis using the RNeasy Plant Mini Kit (Qiagen, USA). One µg of each genomic-digested total RNA sample was reverse transcribed with SuperScript II Reverse Transcriptase (Invitrogen) to synthesize complementary DNA. Quantitative real time-PCR (qRT-PCR) was performed using the iQ SYBR-Green Supermix (Biorad). A linear standard curve was constructed using a serial dilution of cDNA that was pooled from all plants, and generated by plotting the threshold cycle (Ct) against the log10 of the dilution factors. The relative transcript levels of the genes of interest were determined according to the standard curve. *C annuum ACTIN* and *GADPH* genes were used to normalize cDNA concentrations. The primers used are listed in Supplementary Table S1. Data are the means of five biological replicates ± SD.

### Endogenous terpenoids

Semi-polar endogenous metabolites were analysed by LC-Orbitrap-MS using an untargeted metabolomics approach (De Vos et al. 2007). Briefly, 200 mg of leaf material from each sample was extracted with 2 ml 75% MeOH:25% water (HPLC-grade) containing 0.1% formic acid (FA), sonicated for 15 min (40 kHz) and centrifuged for 10 min at 5000×*g*. Ten µl of the supernatant was taken from each sample of the series and combined to make a quality control sample that was analysed every 20 samples to control for machine stability. Metabolites were analysed using a LC-Orbitrap-MS consisting of an Accela U-HPLC equipped with a 1.7 μm AQUITY UPLC BEH C18 column (2.1 × 150 mm; Waters), an Accela photodiode array detector and an ion trap-Orbitrap FTMS hybrid mass spectrometer (Thermo Fisher Scientific) in negative electrospray ionization mode (*m/z* 95–1350) in centroid mode at a source voltage of 4.5 kV. Five µL of each extract was injected and separated with a linear 20 min gradient of 5 to 35% acetonitrile in water (0.1% FA) at a flow rate of 400 µL min^−1^ and 40 °C. The Metalign software (Lommen 2009) was used for baseline correction, mass spectra extraction and mass signal alignment with standard settings. MSClust (Tikunov et al. 2012) was used for data reduction by unsupervised clustering and extracting of putative metabolite mass spectra from ion-wise chromatographic alignment data. Significantly altered metabolites were semi-quantified by comparing characterized ion intensity between non-treated plants and treated plants (Criteria for selection: Fold Change [Treated/Non-treated] > 1.5 or < − 1.5, two-tailed Student’s t test P < 0.05). Putative annotation of non-volatile terpenoid compounds was based on comparison of accurate masses with the KNApSAcK database (http://kanaya.naist.jp/knapsack_jsp/top.html) after correction for possible adducts.

### Volatile terpenoids

Volatiles emitted by plants were collected on Tenax (25/35 mesh) cartridges using a dynamic headspace sampling system as previously described (Zhang et al. 2020). In this system, 12 plants can be sampled simultaneously, and different treated plants were randomized among multiple days while volatiles were collected at the same time of the day (11:00 to 13:00 h). After collection, volatiles were desorbed from the cartridges with a Thermal Desorber TD100-xr (Unity, MARKES International) connected to a GC/Q-ToF MS (7890B GC, Agilent Technologies) and analytes were measured as described in Zhang et al. 2020. Mass spectra were acquired by scanning from 50 to 350 m*/z* with a scan rate of 5 scans min^−1^. Metabolites were identified on basis of chromatographic and spectral comparisons against the NIST11 library (http://chemdata.nist.gov/), Wiley (http://www.sisweb.com/software/wiley-ffnsc.htm), the Adams essential oil library (http://essentialoilcomponentsbygcms.com/) and a comprehensive in-house spectral library generated with authentic standards. For semi-quantification of compounds areas under the curve were calculated and normalized for total leaf area. For each treatment, five replicates were analysed and data show mean ± SD.

### Heterologous expression in *E. coli*

Selected full length *TPS* genes were amplified from cDNA obtained from TSSM-damaged or methyl-JA-treated Capsicum leaves. Primer sequence information is available in Supplementary Table S1. PCR products of open reading frames were cloned into the pACYC-Duet vector and transformed in *E. coli* strain BL21. Single colonies grown in 2 mL LB were induced with 50 µl 1 M IPTG and grown for another 24 h at 18 °C. Subsequently, cells were collected by centrifugation (3.000×*g*), re-suspended in ice-cold extraction buffer (50 mM Tris, pH 7.5, 300 mM NaCl, 1.4 mM β-mercapto ethanol), disrupted using a sonicator (4 times 15 s) and centrifuged (14.000×*g*). The supernatant containing the heterologous protein was used for enzymatic assays. To determine the catalytic activity of TPSs, 150 µl of the crude protein solution was added to 1340 µl assay buffer with 5 μl 10 mM GPP, FPP or GGPP (Sigma-Aldrich) in a screw-capped vial with an overlay of 100 µl pentane and incubated for 1 h at 30 °C with gentle shaking. For analysis of the captured reaction products, the pentane layer was filtered over a Pasteur’s-pipet filled with Na_2_SO_4_ and 2 μL was analysed using GC–MS (5890 series II/5972A, Hewlett-Packard) equipped with a 30 m × 0.25-mm i.d., 0.25 μm film thickness column (5MS, Hewlett-Packard). The GC was programmed at an initial temperature of 45 °C for 1 min, with a ramp of 10 °C min^−1^ to 220 °C and final time of 5 min and a constant Helium column flow of 1.0 mL min^−1^. The injection port, interface, and MS source temperatures were 250 °C, 290 °C, and 180 °C, respectively and the ionization potential was set at 70 eV. Scanning was performed from 45 to 300 atomic mass units. As a negative control, crude protein extract from *E. coli* expressing the empty vector pACYC-Duet incubated with substrate, was used.

One µl of the pentane extract was injected split less at a temperature of 250 °C at an initial column temperature of 40 °C. The temperature was held for 3.5 min, then increased with 10 °C min^−1^ to 280 °C and a hold for 2.5 min at a helium flow of 1 ml min^−1^. The ion source of the DSQ quadrupole mass spectrometer was directly coupled with the analytical column and operated in the 70 eV EI ionization mode. Masses 45 to 350 m*/z* were scanned. Compound identification was performed as described above. For each TPS enzyme and substrate combination, assays were repeated at least once but most often three times.

### Transient expression in leaves of *N. benthamiana*

Plant binary plasmids containing open reading frames of selected TPS were constructed as previously described (Van Herpen et al. 2010). The final and intermediate vectors were checked by enzymatic restriction analysis and sequencing and introduced in *Agrobacterium tumefaciens* AglI by electrotransformation. *A. tumufaciens* cells were infiltrated into the abaxial side of the leaf of *N. benthamiana* plants for transient heterologous expression of CaTPS according to Van Herpen et al (2010). An *A.tumefaciens* strain encoding the TBSV P19 protein was added to suppress gene silencing. In addition, the pIVA_HMGR vector was co-infiltrated to enhance the flux toward terpenoid production. Three days after infiltration, infiltrated leaves were analysed for their volatile emissions and endogenous terpenoid content as described above.

### Statistical analysis

All quantitative data were analysed by Student’s t test and one-way ANOVA using MetaboAnalyst (Version 4.0; www.metabolanalyst.ca). Statistical significance was determined using a Tukey’s honest significant difference.

## Results

### *Capsicum annuum* harbours a mid-size TPS gene family

We identified 103 loci with sequence homology to known *TPS* genes using version 2 of the *C. annuum* genome. The predicted TPS proteins contain one or more TPS characteristic domains, IPR008930, IPR001906, IPR008949, IPR005630 as described in the InterPro database (www.ebi.ac.uk/interpro/) and encode putative proteins with sizes ranging from 102 to 852 amino acids (Supplementary Table S2). Out of the 103 gene models, 27 encode proteins with more than 500 amino acids and are considered to encode functional TPS enzymes.

**Fig. 1:**
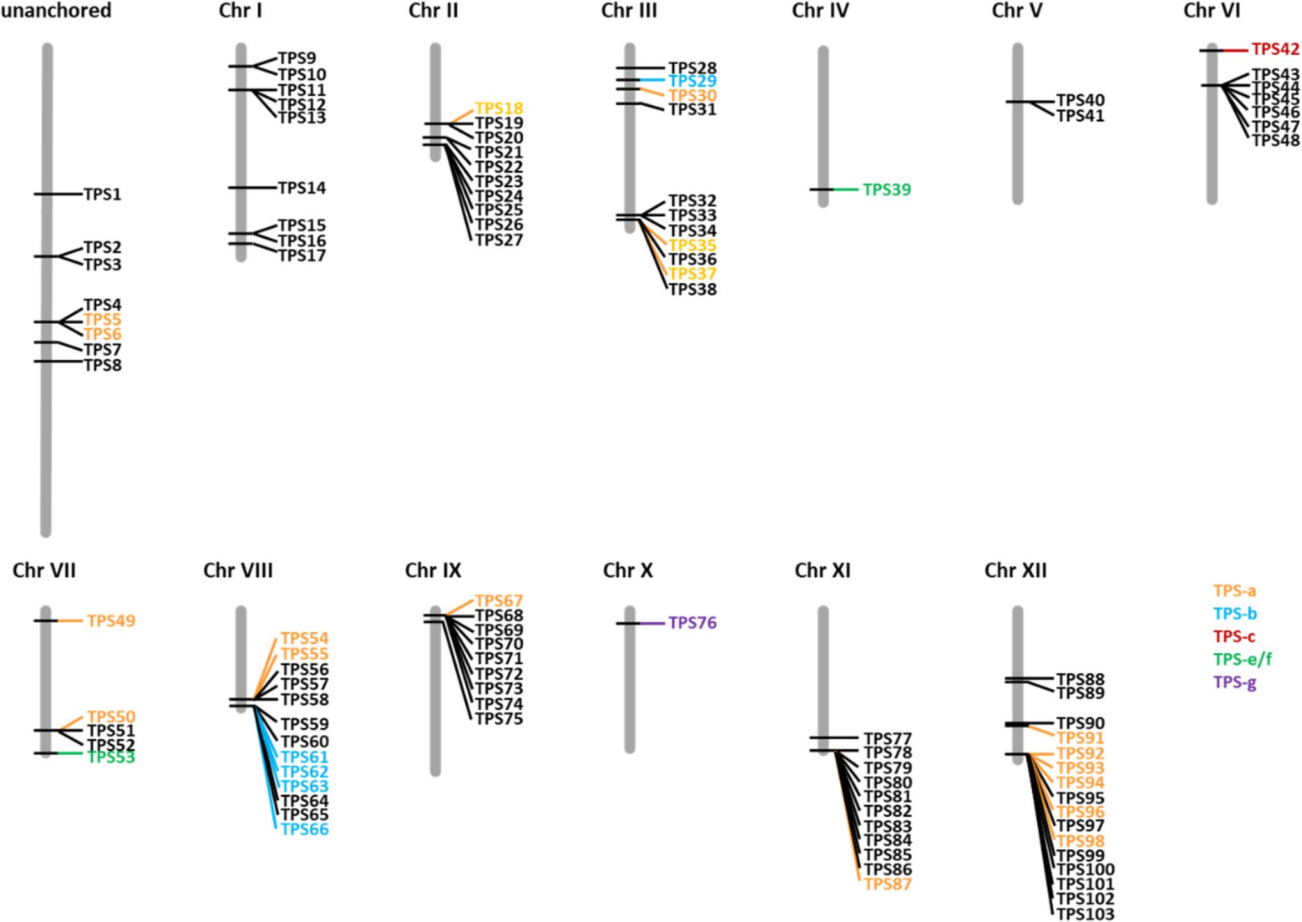
Distribution of *CaTPS* loci in the *Capsicum annuum* genome. The location of all *CaTPS* genes is indicated at the relative position in twelve artificial chromosomes (grey bars). Colour coding is indicative for the TPS sub families: TPS-a, orange; TPS-b, blue; TPS-c, red; TPS-e/g, green and TPS-g, purple

Forty-four of the 103 detected putative Ca*TPS* genes had transcripts present in leaves of which 27 were considered non-functional and 17 putatively functional (Supplementary Table S3). The 103 *TPS* loci are distributed across all 12 chromosomes of Capsicum (Fig. 1), and the majority of these loci are physically located close to each other and show high sequence similarity with each other, suggesting multiple tandem and segmental duplication and recombination events. Such sequential repeat *TPS* gene clusters are located on chromosomes III, VIII, XI and XII, while chromosomes IX, IV, I, VI and VII contain respectively 9, 9, 7, 5 and 2 TPS loci which are more scattered across the chromosome. Chromosome V and X have a single *TPS* locus. Eight *TPS* genes could not be anchored to any specific position and were assigned to an artificial chromosome. In general, *CaTPS* genes are located compactly on the chromosomes with a high tandem repeat structure. Most tandem repeated genes show high similarity in sequence but with diverse gene sizes, suggesting a recent evolutionary tandem duplication of which multiple copies are not complete (anymore).

**Fig. 2:**
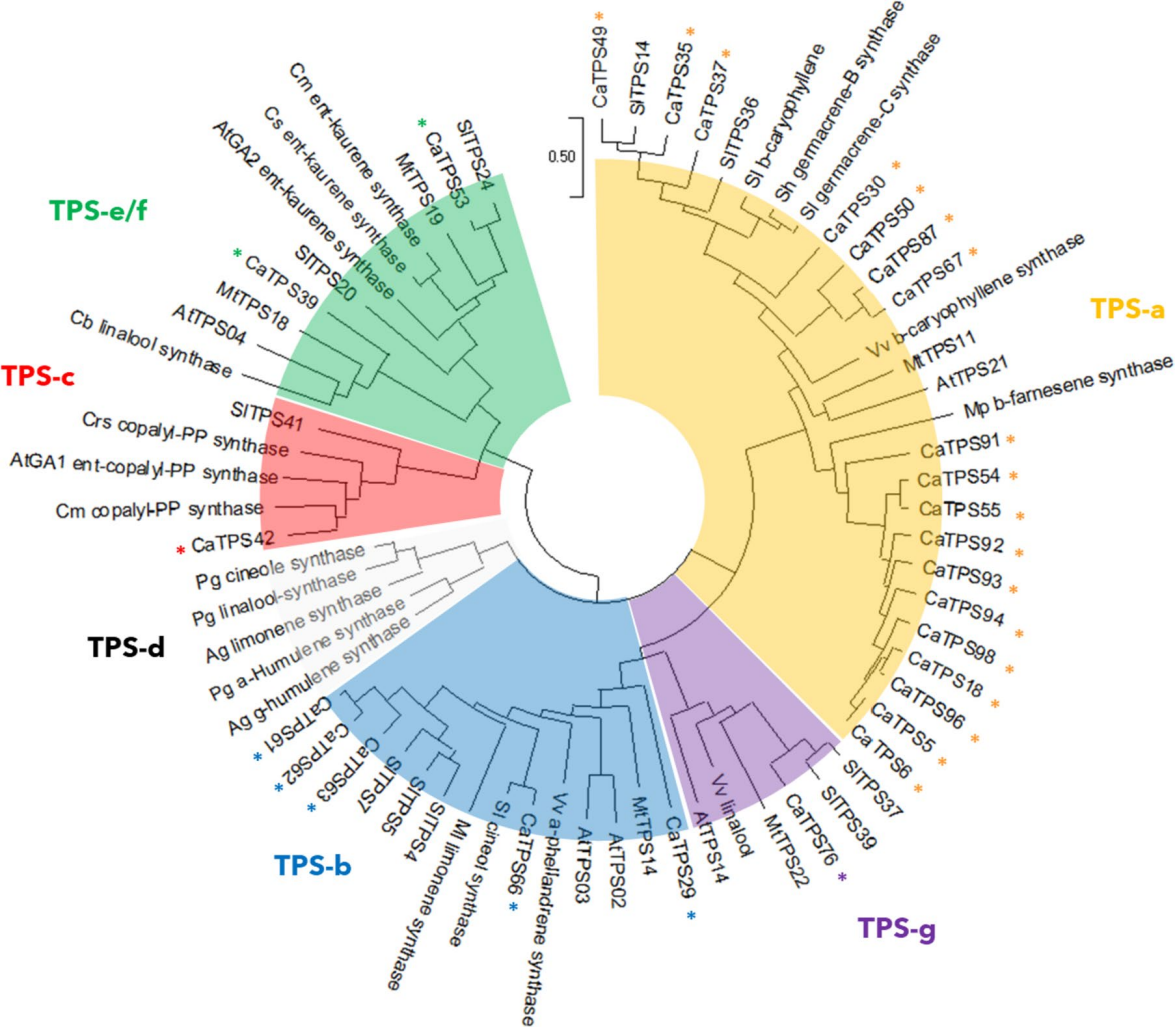
Phylogenetic relationship of full length CaTPS (indicated with *) and selected TPS from other species, representative for each of the TPS sub families. *Ag* *Abies grandis*, *At* *Arabidopsis thaliana*, *Cb* *Clarkia breweri*, *Cm* *Cucumis maximus*, *Crs* *Croton sublyratus*, *Cs* *Cucumis sativus*, *Ml* *Mentha longifolia*, *Mp* *Mentha piperita*, *Mt* *Medicao trunculata*, *Pg* *Picea glauca*, *Sh* *Solanum habrochiates*, *Sl* *Solanum lycopersicum*, *Vv* *Vitis vinifera*. For amino acid sequences and accession numbers, see Supplementary Table S7

Six of the generally recognised eight *TPS* subfamilies are present in the Capsicum genome, with the exceptions being the gymnosperm-specific *TPS-d* subfamily and the *S. moellendorffii*-specific *TPS-h* subfamily (Fig. 2). Most (24 out of 27) *CaTPS* genes belong to class-III type (*TPS-a*: 18 members; *TPS-b*: 5; and *TPS-g*: 1) subfamilies, while class-I *CaTPS* genes comprise a single member for *TPS-c*, *TPS-e* and *TPS-f* each. Gene members from class-III type subfamilies have 6 to 7 exons while the exon number of class-I type subfamily members ranges from 11 to 15, consistent with *TPS* exon numbers found in other species (Trapp and Croteau 2001) (Supplementary Fig. S1).

N-terminal chloroplast transit peptides are predicted for *TPS-b* subfamily members, CaTPS61-63, *TPS-c* members CaTPS42 and *TPS-g* member CaTPS76 (Supplementary Table S4). The presence of a plastid transit peptide in the *TPS-b* members further supports their role as monoterpene synthases. In contrast, no signal peptide was found in the other *TPS-b* members, CaTPS29 and CaTPS66. The putative function as diterpene synthase of the *TPS-c* member CaTPS42 is supported by the presence of a plastid transit peptide. The *TPS-f* member CaTPS39 is also predicted to be a diterpene synthase, but lacks a chloroplast signal peptide. *TPS-g* member CaTPS76 could be either a monoterpene or diterpene synthase.

The conserved RR(X)_8_W motif near the N-terminus, involved in the initiation of the isomerization cyclization reaction or stabilizing the protein through electrostatic interactions (Martin et al. 2010; Aubourg et al. 2002) was present in three out of five putative monoterpene synthases of the *TPS-b* subfamily, while most (16 out of 18) *TPS-a* subfamily members showed variation as (R/P)(S/A/Y/V/I/E)(X)_8_W (Supplementary Fig. S2). The RXR motif was found to be conserved in most CaTPS members and in CaTPS35, CaTPS49, CaTPS53, CaTPS39, CaTPS76 with variation as RX(R/K/Q) but was not conserved in *TPS-c* member CaTPS42. The aspartate-rich DDXXD motif, located about 35 amino acids downstream of the RXR motif and responsible for the divalent metal ion-assisted binding and cleavage of the prenyl diphosphate substrate (Davis and Croteau 2000), is highly conserved in the CaTPS family except for the *TPS-c* member, CaTPS42, a putative copalyl diphosphate synthase (CPS). In Arabidopsis, CPS initiates ionization of GGPP by protonation rather than by diphosphate ester cleavage (Aubourg et al. 2002) (Bohlmann et al. 1998). The aspartate-rich motif (DIDDTA) initiates cyclization by protonation of GGPP (Peters et al. 2001) and is indeed present in CaTPS42 (aa384-389). The (N/D)DXX(S/T/G)XXXE (NSE/DTE) motif is present in all *TPS-b*, *TPS-e/f*, *TPS-g* members and most of the *TPS-a* members but not in *TPS-c* member CaTPS42, nor in three of the TPS-a members CaTPS98, CaTPS91 and CaTPS37. Together with the DDXXD motif, the NSE/DTE motif is also required for metal ion dependent ionization of the prenyl diphosphate substrate which is not present in a monofunctional CPS (Chen et al. 2011).

### Biotic stress affects the terpene biosynthetic module

Using sequence homology, Hidden-Markov-Modeling and genomic context as described by Hofberger et al. (2015), 14 prenyl transferases (PTF), four isopentenyl isomerases (IPP) as well as 18 and 13 proteins predicted to be involved in the MVA and MEP pathways, respectively, were identified in the Capsicum genome, which together with the *CaTPSs* form the terpenoid biosynthetic module (Supplementary Table S3). Furthermore, 15 triterpene synthases were identified.

**Fig. 3:**
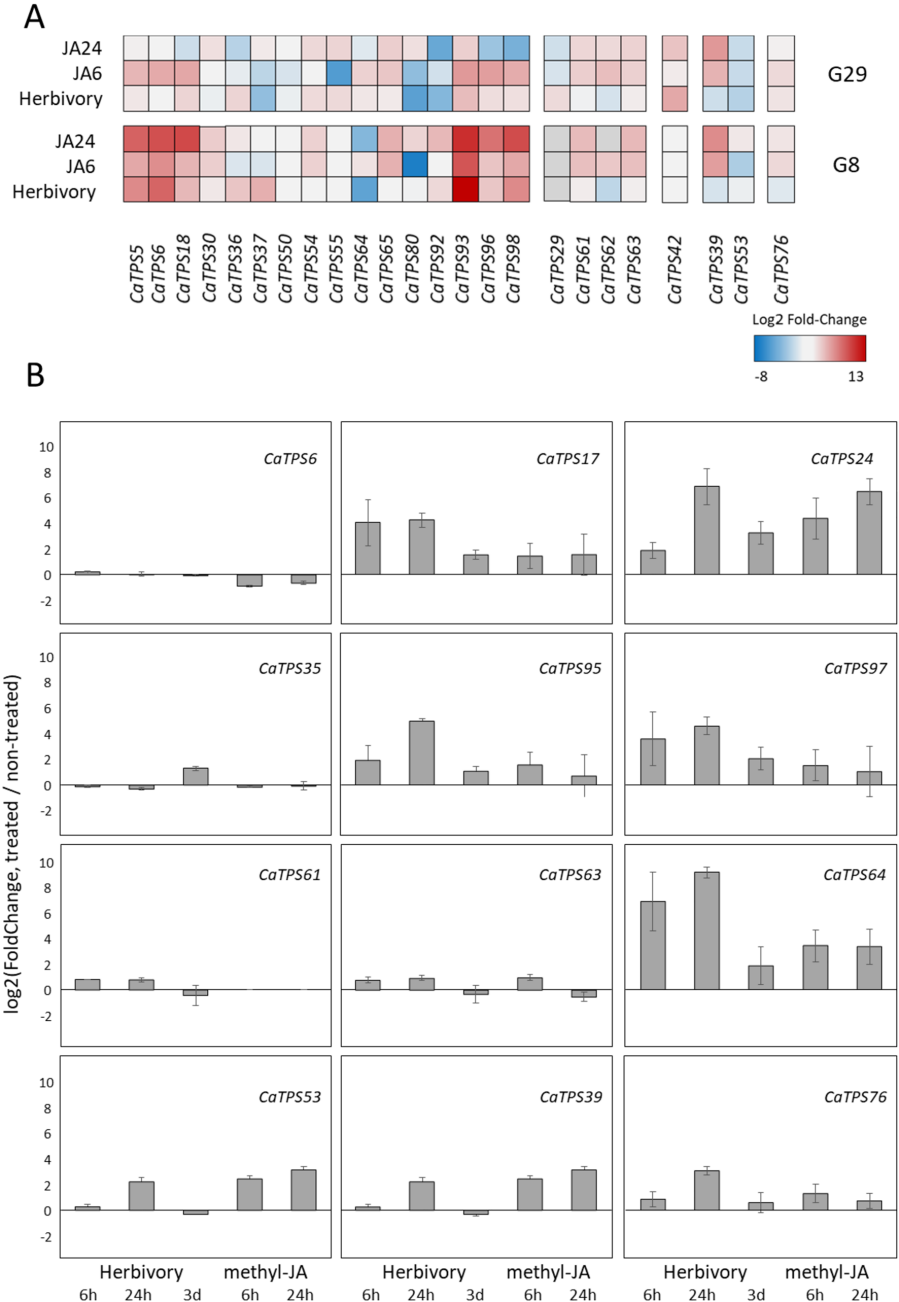
Change in *CaTPS* transcripts as result of spider-mite herbivory or jasmonic acid treatment. **A** heatmap of *TPS* transcripts retrieved from the RNA-seq dataset (Zhang et al. 2020) in which plants were infested with spider mites for three days or treated with JA for 6 or 24 h. Colour coding indicates log2 fold change relative to non-treated leaves. **B** Quantitative RT-PCR analysis of changes in transcripts of selected *TPS* genes relative to non-treated leaves as result of spider-mite infestation for 6 h, 24 h, and three days and methyl-JA treatment for 6 and 24 h. Expression was normalized to the expression of reference genes *CaACTIN* and *CaEF1α.* Data are means of five independent biological replicates ± SD

Expression patterns of genes related to the terpenoid biosynthetic module were examined in two *C. annuum* genotypes that were previously found to differ in spider mite susceptibility, after spider-mite infestation and after JA treatment using a previously generated RNA-sequencing dataset (Zhang et al. 2020). Among the 103 putative *CaTPS* loci, at least 44 transcripts were found present in Capsicum leaves in either genotype or experimental condition. Most *TPS-a* and TPS-*b* subfamily members were induced by spider mites and/or by JA, except *CaTPS49, CaTPS87, CaTPS94* (*TPS-a*) and *CaTPS66 (TPS-b*) (Fig. 3A).

Putative *copalyl diphosphate synthase CaTPS42 (TPS-c)* and *kaurene synthase CaTPS53* (*TPS-e*) were constitutively expressed in Capsicum leaves regardless of genotype or treatment, probably due to its importance for primary metabolite (gibberellin) production, and their expression was slightly altered in opposite directions by spider mites and JA (Fig. 3A, B). Members of the *TPS-f* and *TPS-g* clade, *CaTPS39* and *CaTPS76,* were expressed in non-treated leaves and induced by (methyl)-JA while hardly affected or even slightly repressed by spider mites, dependent of the duration of infestation (Fig. 3A, B).

**Fig. 4:**
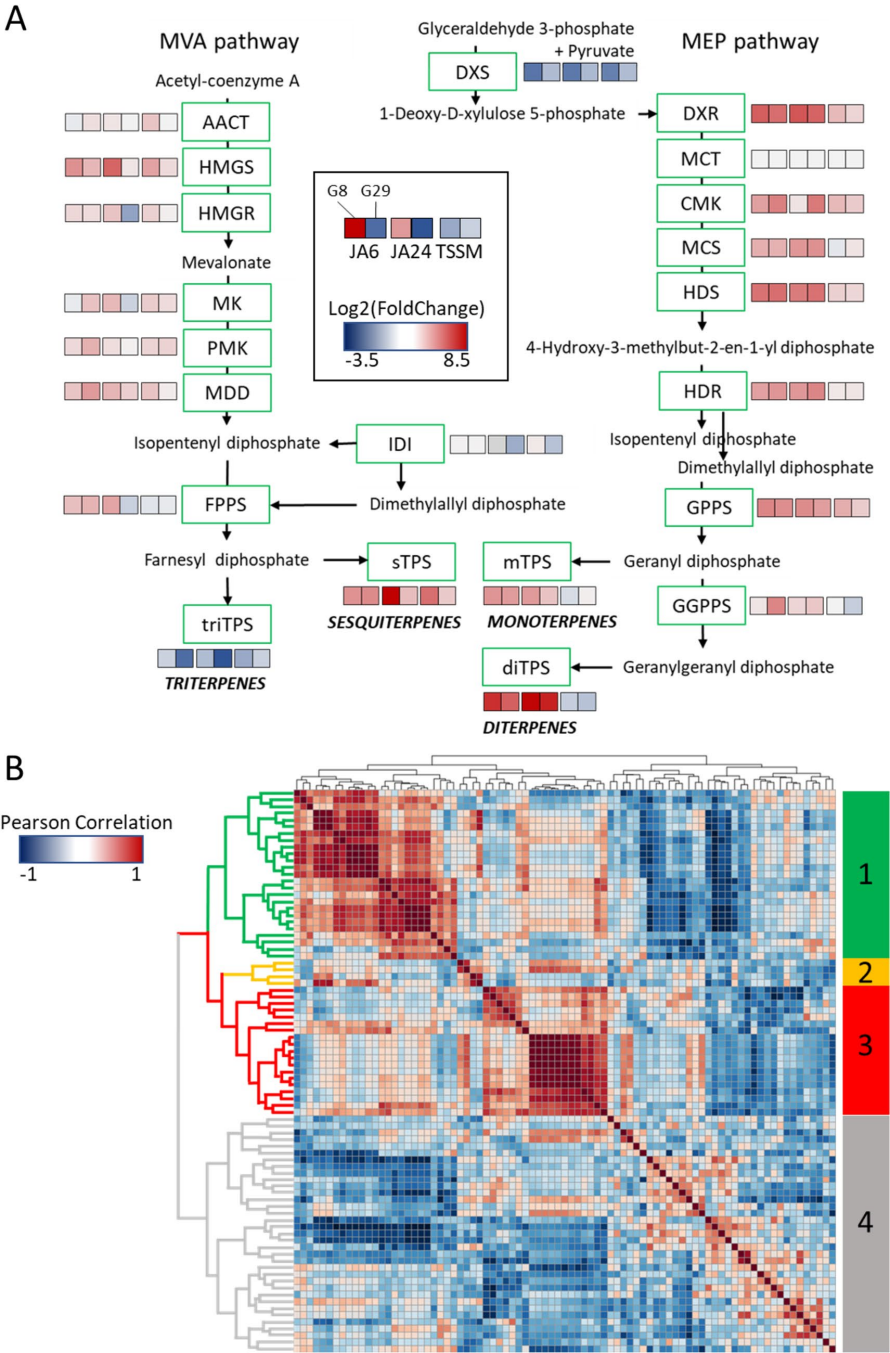
Biotic stress affects the terpenoid biosynthetic module. **A** Expression changes in genes related to the biosynthetic module of the mevalonate (MVA) pathway in the cytosol/peroxisomes and the methylerythritol phosphate (MEP) pathway in the plastids leading to terpenoid precursors; Colour coding indicates log2 fold change relative to non-treated leaves. JA6, JA24: jasmonate treatment 6, 24 h prior to harvest; TSSM: spider-mite herbivory for three days prior to harvest. Gene abbreviations: *AACT* acetyl-CoA acetyltransferase, *HMGS* 3-hydroxy-3-methylglutaryl-CoA synthase, *HMGR* 3-hydroxy-3-methylglutaryl-CoA reductase, *MK* mevalonate kinase, *PMK* phosphomevalonate kinase, *MDD* mevalonate diphosphate decarboxylase, *IDI* isopentenyl-diphosphate delta isomerase, *FPPS* farnesyl diphosphate synthase, *DXS* deoxyxylulose 5-phosphate synthase, *DXR* deoxyxylulose 5-phosphate reductoisomerase, *MCT* 4-diphosphocytidyl-2C-methyl-d-erythritol 4-phosphate synthase, *CMK* 4-diphosphocytidyl-2C-methyl-d-erythritol kinase, *MCS* 2Cmethyl-d-erytrithol 2,4-diphosphate synthase, *HDS* 2C-methylD-erytrithol 2,4-cyclodiphosphate reductase, *HDR* 1-hydroxy-2-methylbutenyl 4-diphosphate reductase, *GPPS* geranyl diphosphate synthase, *GGPS* geranylgeranyl diphosphate synthase, *mTPS* monoterpene synthases, *sTPS* sesquiterpene synthases, *diTPS* diterpene synthases, *triTPS* triterpene synthases. **B** Pearson correlation network showing positive (red) and negative (blue) corelations between genes related to the terpene biosynthetic module, based on their induction profiles in response to biotic stress. Submodule 1 (green) includes multiple genes related to the MEP pathway and putative mono- and diterpene synthases, submodules 2 (orange) and 3 (red) include *TPS-a* clade sesquiterpene *TPS* which correlate with MVA pathway genes in submodule 3. Submodule 4 (grey) includes putative triterpene synthases and various MEP and MVA members. For a high resolution version including gene identifiers see [*add link upon acceptation*]

To obtain insight into how herbivory affects the expression of genes encoding for the MEP and MVA pathways, which provide the substrate for terpene biosynthesis, we calculated the relative expression of all gene candidates in a particular group under each experimental condition (Fig. 4A). All four IPP isomerases, 11 out of 13 MEP pathway genes, 16 out of 18 MVA pathway genes, 12 out of 13 prenyl transferases and 9 out of 15 triterpene synthases were expressed under at least one experimental condition (Supplementary Table S3).

While the expression of genes encoding the MEP pathway was mostly upregulated by JA, this was less pronounced for genes encoding the MVA pathway. Spider-mite infestation resulted in low induction of few genes in the MEP and MVA pathways. Expression levels of IPP isomerases were hardly affected, neither by JA nor by spider mites. Prenyl transferase genes with putative functions such as GPPS or GGPPS were upregulated by JA and spider mites, while in contrast, FPPS genes were upregulated by JA but downregulated by spider mites. Genes with homology to solanesyl diphosphate synthase which are involved in the formation of plastoquinone in the chloroplasts and ubiquinone in the mitochondria were downregulated in all treatments. Together our data suggest that biotic stresses predominantly induce the MEP pathway resulting in increased substrate availability for mono- and diterpene biosynthesis. Indeed, the expression of multiple MEP pathway genes correlated with the expression of putative monoterpene synthases located on chromosome VIII (group 1 in Fig. 4B). In addition, the expression of *TPF-f* subfamily member *CaTPS39* correlated strongly with two putative GGPPS and multiple MEP-pathway genes, supporting their role in diterpene formation.

Interestingly, while most MVA-pathway genes and FPPS were only moderately induced upon JA and not responsive to spider mites, expression of a number of *TPS-a* subfamily members, most likely encoding sesquiterpene synthases, was induced by JA and spider mites. The rate-limiting step in the MVA pathway is considered to be 3-hydroxy-3-methylglutaryl-coenzyme a reductase (HMGR) (Heuston et al. 2012), and indeed, Capana02g000059 and Capana04g002482 both encoding for HMGR are the only genes in the MVA pathway that were upregulated by JA while they were not responsive to spider mites. As the overall expression of triterpene synthases decreased upon JA treatment and spider mite herbivory, possibly substrate was rechannelled towards sesquiterpene biosynthesis resulting in enhanced FPP availability also without upregulation of the MVA pathway and FPP biosynthesis. Finally, there was a, less strong, but clearly visible, correlation between the expression of a number of triterpene synthases and the MEP and MVA pathway genes (group 4 in Fig. 4B). Triterpenes are, however, not within the scope of the present study and are therefore not discussed further.

### Co-expression analysis of TPS genes reveals gene candidates for further diversification of terpenoids

**Fig. 5:**
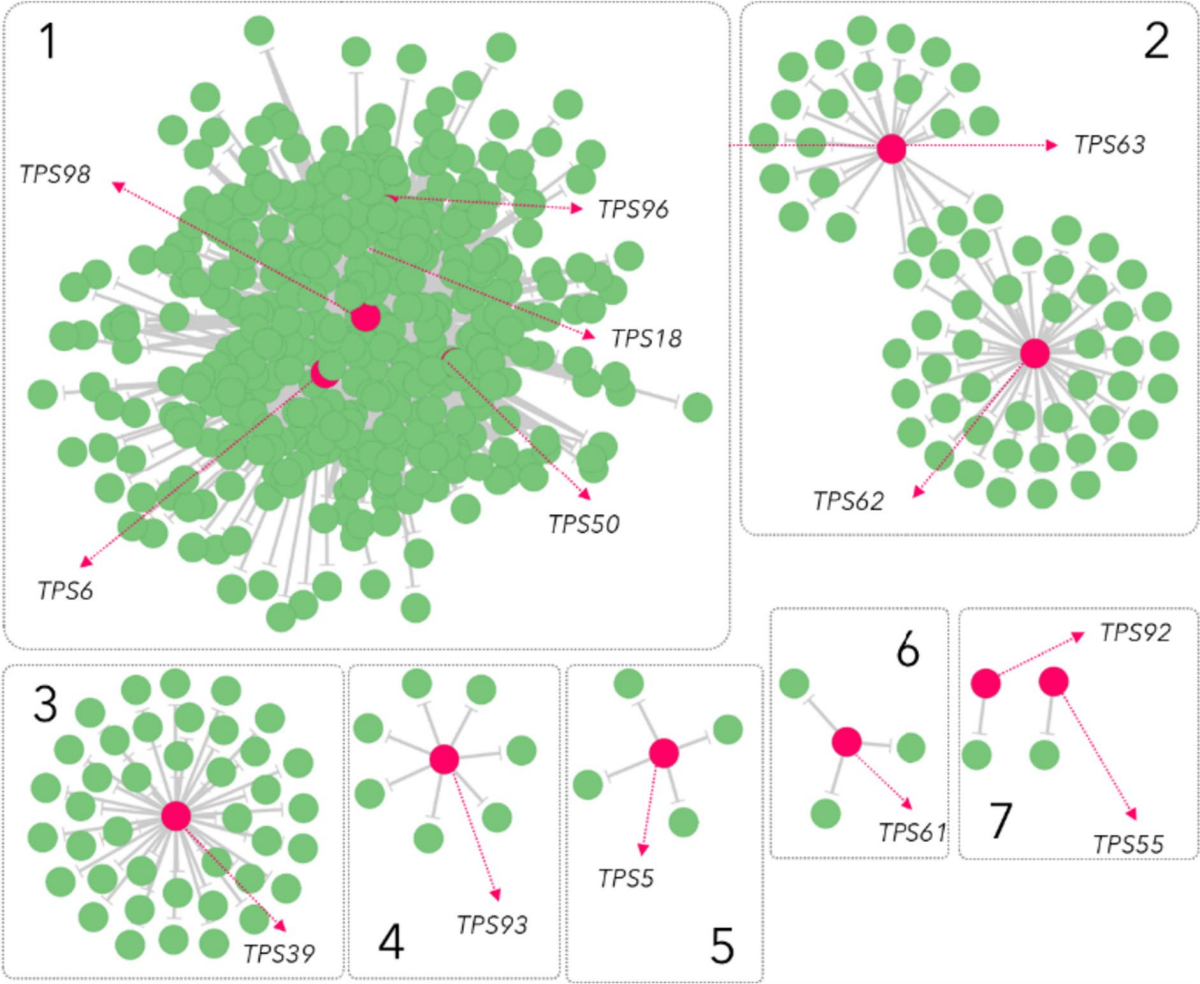
Gene co-expression network of transcripts correlated with *Capsicum annuum TPS* genes that were significantly altered in their expression upon spider-mite infestation or JA induction. Nodes (green) represent genes that show Pearson correlation |r|> 0.9 with TPS genes (magenta). Numbers indicate modules that are addressed in the text. For a detailed figure including gene identifiers based on the Pepper Genome Database (release 2.0) see [*add link upon acceptation*]

To discover other genes possibly involved in Capsicum terpene biosynthesis or its regulation, the expression profiles of 17 expressed and putatively functional TPS genes were used as baits in a co-expression analysis using RNA-seq data (Fig. 5).

Among the expressed TPS genes, 13 showed strong co-expression with a series of other genes resulting in 1606 correlation pairs with 512 genes in total (Supplementary Table S5). The co-expression network showed that TPS genes segregate into distinct sub-networks containing multiple classes of genes. Five *TPS-a* clade genes, *CaTPS6*, *CaTPS18*, *CaTPS50*, *CaTPS96*, *CaTPS98* and their co-expressed genes formed a module (1) with the highest number of nodes and edges). Within this module, *CaTPS18* showed the highest correlation (0.998) with a cytochrome P450, *CYP71D7*. Furthermore, both *CaTPS18* and CaTPS50 displayed high co-expression with multiple putative cytochrome P450s (correlation coefficients varying from 0.974 to 0.988) and *HMGR2* (0.949), encoding the rate-limiting step in the MVA pathway. Co-expressed putative transcription factors included homologues of *WRKY33* (0.985), *WRKY72* (0.983), *ERF2* (0.979), *WRKY75* (0.931) and *MYB4* (0.928), suggesting that *CaTPS18* and *CaTPS50* expression is tightly regulated. Other genes co-expressed with the *TPS-a* clade members mentioned above, include genes putatively encoding galacturonosyl and glutathione transferases.

The highly co-expressed *TPS-b* clade members, *CaTPS62* and *CaTPS63* (correlation coefficient 0.915, module 2), are located closely together on chromosome VIII. This could imply that these genes are regulated by the same transcription factor. Furthermore, several UDP-glycosyltransferase and glutathione S-transferase genes co-expressed with *CaTPS62* and *CaTPS63*. No *WRKY* nor *MYB* transcription factors were found in this module, but a single *ERF113* transcription factor co-expressed (0.938) with *CaTPS62*. In contrast to the module containing *TPS-a* gene members, negative correlations were found for *TPS-b* gene member *CaTPS63* with two putative cytochrome P450s (Capana01g000486, − 0.858; Capana01g000368, − 0.862). The strongest negative correlation with both *CaTPS62* and *CaTPS63* was displayed by Capana08g000288, a putative RNA polymerase sigma factor, which is important to RNA biosynthesis (Ellermeier and Craig 2012).

Positive co-expression relations were found for *TPS-f* member *CaTPS39* with multiple UDP-glycosyltransferases and with genes that likely belong to the JA biosynthetic pathway including a linoleate 13S-lipoxygenase 2-1 (LOX2.1; 0.929) and an allene oxide synthase (AOS; 0.928) in module 3. Furthermore, a putative transcription factor with homology to *AtMYB4*, highly correlated (0.923) with *CaTPS39*. In module 4, *CaTPS93* was found to be correlated with a putative cytochrome P450 (Capana05g001954) and a UTG glucosyl transferase (Capana08g002046), while TPS in modules 5 to 7 correlated mostly to genes with unknown functions.

In conclusion, the co-expression network of Capsicum *TPS* genes that are responsive to spider-mite infestation and/or JA treatment revealed that *TPS-a* subfamily members predominantly co-express with multiple P450s and most of them (except *CaTPS93*) also with MVA pathway gene *HMGR2*. In addition, multiple transcription factors including WRKYs, MYBs, and EFRs correlated with these putative sesquiterpene synthases. Several Capsicum *TPS-a*, *TPS-b* and *TPS-e/f* gene members co-expressed with UDP-glycosyltransferases. Negative gene correlations were predominant among the putative monoterpene synthases suggesting that biosynthesis of monoterpenes is more affected by interaction with other biological processes in plants, while sesquiterpene biosynthesis is more regulated by signalling pathways or external biotic/abiotic stresses.

### Functional characterization of recombinant TPS enzymes

Transcriptional analysis suggests that multiple Capsicum *TPS*s are responsive to biotic stresses. To further characterize the gene functions and their ecological roles in plant defence, nine full-length *TPS* ORFs of genes from Capsicum that were found to be induced by spider mites or JA were expressed in *E. coli* and the recombinant TPS proteins analysed for enzymatic activity (Table 1, Supplementary Tables S6, S9). Consistent with sequence analysis, six TPS-a subfamily members were identified as sesquiterpene synthases as *Ca*TPS6, *Ca*TPS18, *Ca*TPS37, *Ca*TPS96 and *Ca*TPS98 all accepted FPP as substrate to form 4,5-di-*epi*-aristolochene. In addition, *Ca*TPS93 catalysed the conversion of FPP into germacrene A. None of these enzymes accepted GPP nor GGPP, earmarking them as monofunctional sesquiterpene synthases. TPS-f subfamily member *Ca*TPS39 was characterized as a bifunctional enzyme accepting both FPP and GGPP to produce (*E*)-nerolidol and geranyl linalool, respectively, while no product was detected using GPP as substrate. Except for *Ca*TPS63, expressed TPS-b subfamily members could not be cloned or characterized. *Ca*TPS63 accepted GPP to produce β-pinene. TPS-g member *Ca*TPS76 accepted both GPP and FPP to form linalool and (*E*)-nerolidol, respectively, while no product was detected using GGPP as substrate.

**Table 1:** Functional in vitro characterization of selected recombinant CaTPS proteins upon incubation with geranyl diphosphate (GPP), farnesyl diphosphate (FPP) and geranyl geranyl diphosphate (GGPP)

CaTPS	Accepted Substrate	Major product	Molecular Formula	Biochemical Class
		Formed		
*Clade a*				
TPS6	FPP	4,5-di-*epi*-Aristocholene	C_15_H_24_	Sesquiterpene
TPS18	FPP	4,5-di-*epi*-Aristocholene	C_15_H_24_	Sesquiterpene
TPS37	FPP	4,5-di-*epi*-Aristocholene	C_15_H_24_	Sesquiterpene
TPS93	FPP	Germacrene A	C_15_H_24_	Sesquiterpene
TPS96	FPP	4,5-di-*epi*-Aristocholene	C_15_H_24_	Sesquiterpene
TPS98	FPP	4,5-di-*epi*-Aristocholene	C_15_H_24_	Sesquiterpene
*Clade b*				
TPS63	GPP	β-Pinene	C_10_H_16_	Cyclic monoterpene
*Clade f*				
TPS39	FPP	Nerolidol	C_15_H_26_O	Sesquiterpene alcohol
	GGPP	Geranyl linalool	C_20_H_34_O	Diterpene alcohol
*Clade g*				
TPS76	GPP	Linalool	C_10_H_18_O	Acyclic monoterpene alcohol
	FPP	Nerolidol	C_15_H_26_O	Acyclic monoterpene alcohol

Enzymes were incubated with all substrates, but only successful product formations are presented

Transient in-planta overexpression of the abovementioned TPS in *N*. *benthamiana* confirmed the results of the in-vitro assays, although more terpenoid products were detected that were not present in the *E. coli* assays or in trace amounts (Supplementary Table S6).

### Induced terpenoids in Capsicum in response to biotic stresses

**Fig. 6:**
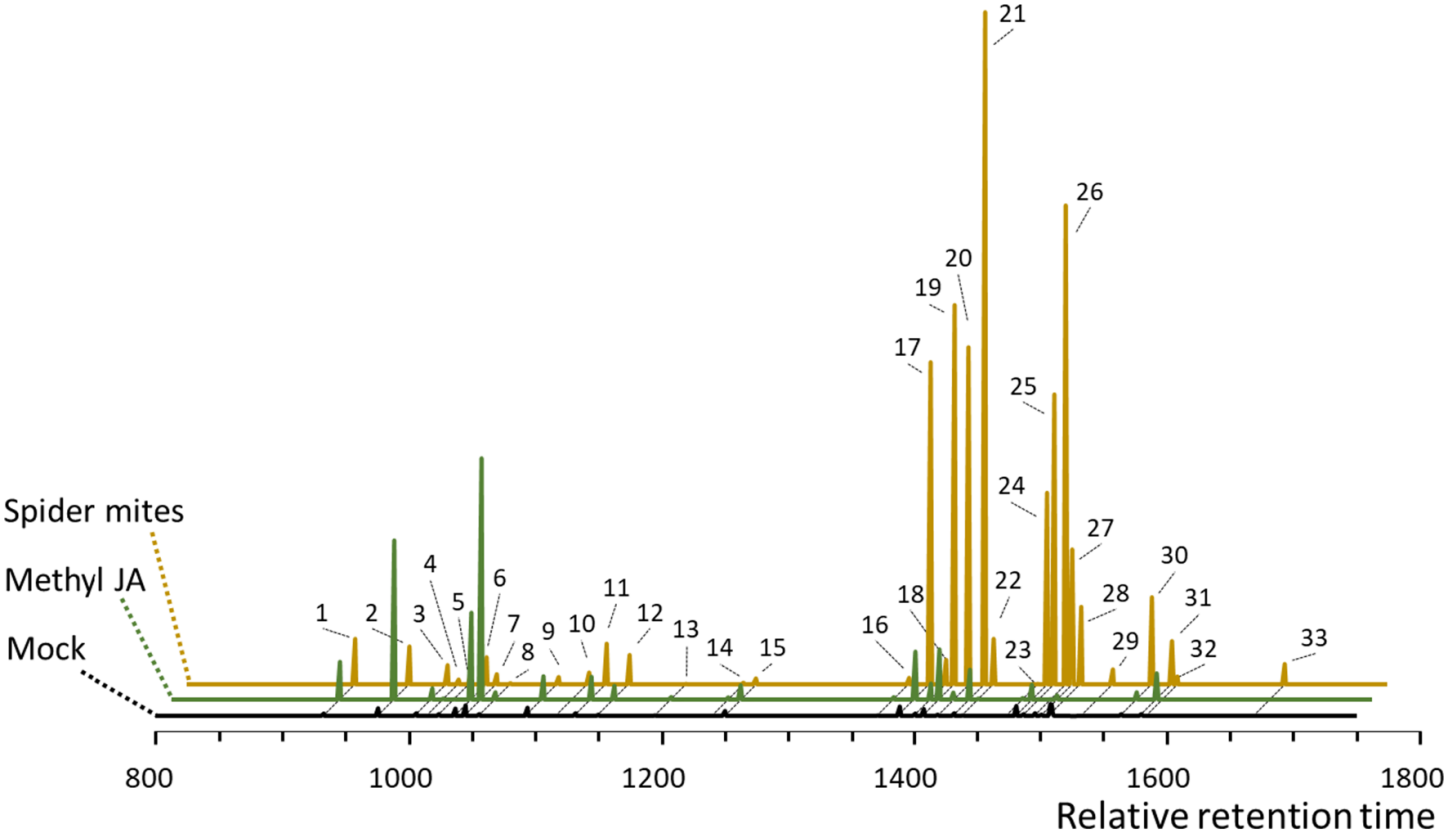
Emitted volatile terpenoid metabolites from *Capsicum annuum* leaves that were infested with spider-mites (brown), treated with methyl-jasmonate (green) or not treated (black). Data show GC–MS area units at the relative retention time. Numbers indicate compound (Retention Index); 1, α-Pinene (933); 2, β-Pinene (976); 3, 3-carene (1006); 4, Unknown monoterpene (1015); 5, Limonene (1024); 6, (Z)-β-Ocimene (1037); 7, (E)-β-Ocimene (1045); 8, γ-Terpinene (1056); 9, Linalool (1094); 10, (E)-DMNT (1118); 11, (E)-β-Ocimene epoxide (1132); 12, Neo-allo-ocimene (1150); 13, Unknown monoterpene alcohol (1195); 14, 3-Caren-2-one (1240); 15, Geraniol (1250); 16, α-Copaene (1372); 17, β-cubebene (1389); 18, β-Longipinene (1401); 19, (Z)-α-Bergamotene (1408); 20, β-Caryophyllene (1419); 21, (E)-α-Bergamotene (1432); 22, Aromandendrene (1439); 23, γ-Muurolene (1474); 24, γ-Himachalene (1481); 25, β-Selinene (1487); 26, α-Selinene (1496); 27, Unknown sesquiterpene (1501); 28, Germacrene A (1508); 29, (Z)-Nerolidol (1533); 30, (E)-Nerolidol (1564); 31, Caryophyllene oxide (1580); 32, (E,E)-TMTT (1584); 33, Unknown sesquiterpene lactone (1688)

To characterize terpenoid metabolites produced upon biotic stress, both volatile and endogenous terpenoids were analysed in (the headspace of) leaves of two genotypes in response to spider mite herbivory and methyl-JA treatment. At least 34 terpenoids were detected in the volatile blend emitted by non-infested (Mock-treated) plants, of which the dominant compounds were monoterpenes (*E*)-β-ocimene and β-pinene, and sesquiterpene germacrene A in both genotypes (Fig. 6). While the abundance of specific monoterpenoids increased in response to spider-mite feeding, the overall contribution of monoterpenoids decreased due to the stronger increase in abundance of multiple sesquiterpenoids and homoterpenes DMNT and (*E,E*)-4,8,12-trimethyltrideca-1,3,7,11-tetraene (TMTT).

Furthermore, spider mites and methyl-JA altered the abundance of endogenous and mostly glycosylated terpenoids (Table 2). Abundance of tetraterpenoid carotenoids, including β-cryptoxanthin and zeaxanthin decreased in response to spider-mite feeding and methyl-JA treatment. Most endogenous terpenoids that increased in response to spider-mite infestation and/or methyl-JA are putative hemi- and diterpene glycosides. Furthermore, a number of triterpene saponins and steroid terpenoids conjugated with glycosides were found to be mostly induced in genotype 29.

**Table 2:** Non-volatile terpenoid compounds in leaves of two *Capsicum annuum* genotypes of which abundances are altered upon spider-mite infestation or JA treatment

Biochemical class	Molecular Formula	Parental ion [M-H]^−^	log2(Fold Change) relative to non-induced leaves			
			Genotype 8		Genotype 29	
			JA	Spider mites	JA	Spider mites
Hemiterpene glycosides	C35H38O17	729.2321	0.94	–	0.61	–
Diterpene glycosides	C26H38O11	525.2443	–	–	>>	–
	C44H70O22	949.4248	− 2.41	–	–	<<
Triterpene Glycosides	C57H88O25	1171.5306	–	–	2.04	1.85
	C34H54O4	525.4871	–	–		>>
	C45H72O16	867.8970	− 0.97	–	–	–
Triterpene saponins	C57H90O25	1173.5346	–	–	1.86	1.78
	C51H78O20	1009.4779	–	–	2.64	1.79
	C57H90O25	1173.5346	–	–	1.80	–
	C59H92O26	1215.5352	–	–	0.78	–
	C51H78O20	1009.4779	–	–	1.59	–
Tetraterpene carotenoids	C40H56O	651.1578	<<	–	− 1.89	<<
	C40H56O2	567.2094	− 0.89	–	− 2.04	–
	C40H56O3	583.2772	–	–	0.85	–
Steroid Glycosides	C54H84O25	1131.5353	–	–	1.32	1.74
	C48H74O20	969.4824	–	–	2.35	1.68
	C30H44O6	499.2573	2.54	2.09		

Putative annotation in compound class was done by comparing accurate masses with the KNApSAcK database. Metabolites analysis was based on three replicates of each sample (Student-T Test with equal variation; P < 0.05, FoldChange > 1.5 or < − 1.5). ‘>>’, not present in non-induced leaves; ‘<<’, absent in induced leaves; ‘–’ abundance was not significantly different

## Discussion

Terpenoids are the most widely occurring secondary metabolites in nature and show enormous diversity across the plant kingdom. Many of them play important roles in plant direct or indirect defence against microbes and herbivores (Gershenzon and Dudareva 2007; Pichersky and Raguso 2018). Here, we aimed to increase our understanding of how the terpenoid biosynthetic pathway in the economically important crop species *Capsicum annum* is affected by cell-content feeding spider mites. We used a combinatorial approach to identify the Capsicum *TPS* gene family and the genes responsible for the generation of precursors and those that might be involved in further terpenoid diversification. Our results provide insight into Capsicum specialized terpenoid metabolism related to biotic stress and provide a template for further exploration of genetic resources for Capsicum breeding for improved resilience against herbivores.

Compared to *TPS* gene families of other species, the *CaTPS* gene family is of moderate size and characterized by a significant number of pseudogenes and short gene fragments. For instance, in *P. patens* (Hayashi et al. 2007), there is one full-length gene out of four gene models, while Arabidopsis (Aubourg et al. 2002) has 32 full-length genes out of 40 gene models. Additionally, apple contains 10 full-length genes out of 55 gene models (Nieuwenhuizen et al. 2013), tomato 29 full-length genes out of 44 gene models (Falara et al. 2011; Zhou and Pichersky 2020) and grape contains 69 full-length genes out of 152 gene models (Martin et al. 2010). This is probably due to a relatively high speed of duplication accompanied by gene degradation and loss of function. *TPS-a* and *TPS-b* subfamilies form the two largest clades in the *CaTPS* family and most of the genes in these clades have high sequence homology and closely co-localize on the chromosomes. This clustered localization is prominent for *TPS-a* members on chromosome II, III, VIII, IX, XI, XII and for *TPS-b* members on chromosome VIII. Many TPS genes of the *TPS-a* and *TPS-b* clade have likely resulted from local duplication events (Aubourg et al. 2002). Those gene copies tend to be retained as their products are specialized metabolites that will not affect the development and survival of the plant. Hence, this high speed of duplication may not be restricted too much by selection pressure (Jiang et al. 2019; Heidel-Fischer and Vogel 2015; Pichersky and Gang 2000). The rapid evolution of TPS genes has resulted in species–specific paralogous TPS gene clusters. Despite the high frequency of duplication and gene degradation, all *TPS-a* family members have conserved DDXXD and RXR domains. The angiosperm-specific *TPS-a* subfamily is highly divergent across all the seed plants and *TPSs* in this clade are either sesquiterpene synthases or diterpene synthases while *TPS-b* family members mainly encode cyclic monoterpene and hemiterpene synthases (Chen et al. 2011).

In contrast to the abovementioned subfamilies, *TPS* genes from the *TPS-c*, *TPS-e/f* and *TPS-g* clades are maintained as a single copy. The low ratio of divergence and slow speed of duplication indicate a strong selection. Sequence analysis suggested that *CaTPS53*, the only member in the *TPS-e* clade, encodes *ent*-kaur-16-ene synthase. Together with *CaTPS42* (*TPS-c*) with high homology to copalyl diphosphate synthase, these genes are probably responsible for the production of gibberellin in Capsicum (Prisic et al. 2004; Xu et al. 2007).

Terpene biosynthesis depends on the activity of various independent pathways acting in balance to convert primary metabolites to specialized compounds designed to mediate interactions of plants with their surroundings. The modular organization of plant terpenoid biosynthesis includes distinct reaction modules, including genes from the MVA and MEP pathways, IPP isomerases, prenyl transferases and TPS genes encoding monoterpene, sesquiterpene and diterpene synthases (Hofberger et al. 2015). Furthermore, triterpene synthases are considered to be part of the terpenoid biosynthetic module and they share a common evolutionary origin with genes from the MVA and MEP pathway (Matsuba et al. 2013). We were able to identify gene candidates for each of the reaction modules leading to the substrates for terpene synthases, and the expression of these genes correlated with *TPS* expression, implying that the biosynthetic module is regulated in response to biotic stress.

The plastidial MEP pathway is the supplier for primary metabolites important in photosynthesis and hence of major relevance for growth. Herbivory is known to induce defences with often a trade-off to growth. Miltra et al. (2021) demonstrated that *Spodoptera littoralis* herbivory on Arabidopsis decreased flux through the MEP pathway via DXP and DXS inhibition. In Capsicum, spider-mite herbivory suppressed terpenoid biosynthetic flux through genes in both the MVA and the MEP module as well as the IPP and PTF modules, while the effect of jasmonate was mostly apparent through a decrease in gene expression in the IPP module. The down-regulation of the MEP pathway relates with a decreased abundance of metabolites derived from the plastids, including volatile monoterpenoids, and endogenous terpenoid-glycosides and carotenoids. The latter might partly explain the suppression of photosynthesis in response to herbivory (Zangerl et al. 2002) and indeed, our RNA-sequencing data showed lower transcript abundances of genes related to photosynthesis (Zhang et al. 2020). In our current work, we aim to characterize the role of MEP and MVA pathway genes in Capsicum in relation to the growth-defence trade-off, in order to predict the impact of cell-content feeders on Capsicum specialized metabolism and growth.

Out of 27 predicted full length TPS genes, we selected nine genes for heterologous expression. The terpene synthases that were selected from the TPS-a subfamily displayed high homology with 5-*epi*-aristolochene synthase, and indeed five of them produced 4,5-di-*epi*-aristolochene, an isomer of 5-*epi*-aristolochene. While the expression of these genes was clearly induced upon spider-mite infestation or JA-treatment, 4,5-di-*epi*-aristolochene was not detected in the headspace of the two genotypes. 5-*Epi*-aristolochene is the precursor for the production of the anti-microbial sesquiterpenoid capsidiol which is produced upon stress in several Solanaceous species (Whitehead et al. 1989; Bohlmann et al. 2002; Lee et al. 2017; Zavalapáramo et al. 2000). The oxidation of 5-*epi*-aristolochene to capsidiol has been shown to be mediated by elicitor-inducible cytochrome P450 hydroxylase, CYP71D in tobacco (Ralston et al. 2001). In Capsicum, the expression of *CaTPS18* and *CaTPS98* exhibited a high correlation with cytochrome P450s annotated as CYP71 family members, which are thus likely candidates for the oxidation of 4,5-di-*epi*-aristolochene. Upon spider-mite infestation and JA-treatment, a compound with a similar accurate mass as capsidiol, but different retention time, was found in genotype 8, suggesting that in Capsicum 4,5-di-*epi*-aristolochene is converted into a structural homolog of capsidiol (Supplementary Fig. S3).

Except *CaTPS29*, CaTPS-b subfamily members have the conserved RRW8R domain and three of them were predicted to have a transit peptide which would direct the proteins to the chloroplasts. Only the genes predicted to have a transit peptide were found to have transcripts in the RNA-seq data and they show high similarity to a (−)-camphene/tricyclene synthase from *C. baccatum* (Kim et al. 2017). The headspace of both *C. annuum* genotypes contained at least 14 different monoterpenoids of which the abundance of 8 altered in response to spider-mite infestation. We were able to successfully characterize CaTPS66, which accepts GPP to form the cyclic monoterpene, β-pinene in-vitro and in-planta when transiently expressed in *N. benthamiana*, and which shares a 92.7% similarity with a monoterpene synthase from tomato (*Sl*TPS8) catalysing the formation of cyclic 1,8-cineole from both GPP and NPP (Falara et al. 2011).

TPS-e member *Ca*TPS53 shows 94.4% similarity with *Sl*TPS24 which is a kaurene synthase (Falara et al. 2011) and *Ca*TPS39 in the TPS-f clade is closely related to *At*TPS04 ((*E,E*)-geranyl linalool synthase), *Mt*TPS18 ((*E*)-nerolidol/(*E,E*)-geranyl linalool synthase) and *Cb*S-linalool synthase (Aubourg et al. 2002; Parker et al. 2014; Dornelas and Mazzafera 2007).

TPS-f member *Ca*TPS39 catalyses both the formation of (*E*)-nerolidol and (*E,E*)-geranyl linalool, and its transcripts were induced by both spider mites and JA. Both (*E*)-nerolidol-derived DMNT and (*E,E*)-geranyl linalool-derived TMTT are detectable in the Capsicum spider-mite induced volatile blend. *CaTPS39* expression correlates well with the expression of a putative *CYP82* (Capana04G000466), which is localized in the proximity of CaTPS39 on chromosome IV, suggesting that this cytochrome P450 could be responsible for the conversion of geranyl linalool to TMTT. A CYP82 member was shown to convert geranyl linalool into DMNT and TMTT, in *Zea mays* and Arabidopsis (Richter et al. 2016; Lee et al. 2010). Expression of a (*E*)-nerolidol/(*E,E*)-geranyl linalool synthase in Arabidopsis resulted in enhanced emission of (*E*)-nerolidol, DMNT and TMTT, and improved attraction of parasitoid wasps, *Diadegma semiclausum* (Houshyani et al. 2013) as well as predatory mites *Phytoseiulus persimilis* (Kappers et al. 2005). The presence of TMTT in the headspace of Capsicum upon spider-mite feeding is likely due to the concerted expression of TPS39 and CYP82 gene candidates. TPS-g clade member, *Ca*TPS76 encodes a monoterpene synthase catalysing the conversion of GPP to acyclic linalool and also accepts FPP as substrate to produce acyclic (*E*)-nerolidol. *Ca*TPS76 has high sequence similarity with *Sl*TPS37 and *Sl*TPS39 from tomato with similarities of 89.0% and 86.5% respectively, catalysing the formation of linalool and (*E*)-nerolidol from GPP and (*E,E*)-FPP, respectively (Falara et al. 2011). Multi-substrate accepting enzymes capable to catalyse both the formation of monoterpenes and sesquiterpenes have been reported in multiple species (Degenhardt et al. 2009) and linalool/nerolidol synthases have been identified in Arabidopsis (Chen et al. 2011), tomato (Falara et al. 2011), cucumber (He et al. 2022) and grape (Zhu et al. 2014). In Capsicum, the ratio between linalool and nerolidol in the volatile blend differed when leaves were biotically challenged, which might be explained by the altered flux of nerolidol towards DMNT in response to herbivory. However, we did not detect any highly correlated genes for CaTPS76 possibly involved in the conversion of nerolidol to DMNT.

Genes encoding biosynthetic enzymes for specialized metabolites are often located physically close on chromosomes, forming clusters in the plant genome, such as the biosynthetic genes responsible for the formation of triterpenoid limonoids in citrus and neem (De La PeÑa et al. 2023), petirucalla-7,24-dien-3b-ol in Arabidopsis (Boutanaev et al. 2015), cucurbitacin C in *Cucumis sativus* (Shang et al. 2014) and diterpenoid casbene in *Euphorbia peplus* (King et al. 2014). Combinations of terpenoid synthases with cytochromes P450s are found clustered together in plant genomes far more commonly than would be expected by chance and this gene pairing likely has evolved from a common ancestral pairing in eudicots (Boutanaev et al. 2015). Many metabolites formed as result of genes within such plant metabolic gene clusters appear to provide protection against pathogens and herbivores. Clustering genes and coinheritance of optimized gene combinations that confer selective advantage is probably beneficial for plants (Yeaman and Whitlock 2011; Takos and Rook 2012). Besides, co-localization of metabolic pathway genes increases the likelihood of co-regulating gene expression and prevents the accumulation of auto-toxic intermediates (Field et al. 2011; Xu et al. 2012).

In Capsicum, multiple genes including cytochromes P450s, prenyl transferases, and MVA and MEP pathway genes were detected to localise physically close to TPS genes, suggesting specialized gene-clusters putatively under the control of a shared regulatory mechanism as many of them are co-expressed upon biotic stress. Especially members of the TPS-a subfamily tend to cluster with cytochrome P450 genes. The clustering of *TPSs* with glycosyl transferases and with cytochrome P450s matches with the findings that multiple monoterpene glycosides, oxygenated sesquiterpenes, diterpene glycosides and triterpene glycosides increased upon spider-mite feeding and/or JA treatment.

In conclusion, we identified the *TPS* gene family in the Capsicum genome and analysed their phylogeny, gene structure and expression profile upon spider-mite feeding and JA treatment. Upstream biosynthetic genes from the MEP and MVA pathway and downstream modification genes including cytochrome P450s, UDP-glycosyl transferases and transcription factors that under herbivory display co-expression with *CaTPS* genes were identified. Finally, recombinant proteins of selected TPSs were functionally characterized. Together, our work demonstrates the importance of the terpenoid biosynthetic network for the formation of volatile and non-volatile defence-related terpenoids in *Capsicum annuum*.

### Supplementary Information

Below is the link to the electronic supplementary material.

Supplementary file1 (XLSX 1626 KB)

## Data Availability

RNA-sequencing data are available at Dryad Data Repository: 10.5061/dryad.n34h180. Metabolomics data files (LC–MS, GC–MS) will be deposited in Dryad upon acceptance of the manuscript.
